# Downscaling the Sample Thickness to Sub-Micrometers by Employing Organic Photovoltaic Materials as a Charge-Generation Layer in the Time-of-Flight Measurement

**DOI:** 10.1038/srep10384

**Published:** 2015-05-22

**Authors:** Shun-Wei Liu, Chih-Chien Lee, Wei-Cheng Su, Chih-Hsien Yuan, Chun-Feng Lin, Kuan-Ting Chen, Yi-Sheng Shu, Ya-Ze Li, Tsung-Hao Su, Bo-Yao Huang, Wen-Chang Chang, Yu-Hsuan Liu

**Affiliations:** 1 Department of Electronic Engineering, Ming Chi University of Technology, New Taipei City 24301, Taiwan; 2 Department of Electronic Engineering, National Taiwan University of Science and Technology, Taipei 10607, Taiwan

## Abstract

Time-of-flight (TOF) measurements typically require a sample thickness of several micrometers for determining the carrier mobility, thus rendering the applicability inefficient and unreliable because the sample thicknesses are orders of magnitude higher than those in real optoelectronic devices. Here, we use subphthalocyanine (SubPc):C_70_ as a charge-generation layer (CGL) in the TOF measurement and a commonly hole-transporting layer, N,N’-diphenyl-N,N’-bis(1,1’-biphenyl)-4,4’-diamine (NPB), as a standard material under test. When the NPB thickness is reduced from 2 to 0.3 μm and with a thin 10-nm CGL, the hole transient signal still shows non-dispersive properties under various applied fields, and thus the hole mobility is determined accordingly. Only 1-μm NPB is required for determining the electron mobility by using the proposed CGL. Both the thicknesses are the thinnest value reported to data. In addition, the flexibility of fabrication process of small molecules can deposit the proposed CGL underneath and atop the material under test. Therefore, this technique is applicable to small-molecule and polymeric materials. We also propose a new approach to design the TOF sample using an optical simulation. These results strongly demonstrate that the proposed technique is valuable tool in determining the carrier mobility and may spur additional research in this field.

Organic materials enabled fabricating optoelectronic devices onto large-area and flexible substrates in low-cost approaches^1^,^2^,^3^,^4^,^5^. The applications such as organic light-emitting diodes (OLEDs), organic photovoltaic (OPV) devices, and organic thin-film transistors (OTFTs), are greatly progressing toward the next-generation organic electronics because device performance considerably improves^6^,^7^,^8^,^9^,^10^,^11^,^12^,^13^,^14^,^15^. Unfortunately, the inherent nature of organic materials (i.e., the amorphous molecular structure) restricts the maximum performance of these devices because of the relatively low carrier mobility compared with their inorganic counterparts. Therefore, developing novel materials is essential to improve both the optical and electronic properties^16^,^17^,^18^,^19^,^20^,^21^. In particular, carrier mobility is of special interest for the electronic properties because of its great impact on the electrical transport, carrier distribution, carrier recombination, charge separation, and charge balance condition^22^,^23^,^24^,^25^,^26^,^27^,^28^,^29^,^30^,^31^,^32^,^33^. Because these factors determine the device performance, an in-depth understanding and detailed analysis are required for characterizing the properties of carrier transport.

The carrier mobility can be characterized using time-of-flight (TOF) measurement, space-charge limited current (SCLC) model, transient electroluminescence (EL) measurement, and field-effect transistors (FETs)^34^,^35^,^36^,^37^. The mobility evaluation through FETs provides an easy and low-cost approach because of a simple device structure. However, the general geometry of FETs in which the current flows parallel to the substrate may lead to an undesirable result for the OPV devices and the OLEDs because the electrical properties in these devices are primarily dominated by the currents perpendicular to the substrate^38^,^39^. On the contrary, the SCLC estimates the carrier mobility along the vertical direction with respect to the substrate^40^. Previous reports have successfully evaluated the carrier mobility of common materials in OLEDs^41^,^42^. Although the values are in good agreement with other measurements, a careful and proper selection of electrode for achieving Ohmic contact is essential to satisfy the fundamentals of the SCLC model. Therefore, the SCLC may show a highly material-dependent restriction. The transient EL measurement use the time delay between the driving voltage pulse and the peak of EL emission for determining the carrier mobility^36^. This technique was improved over the last decade through varying the film thickness and device area for considering the charging effect at the interface and averaged electric field across the device, thus permeating a comparable estimation for the intrinsic property of materials^43^,^44^. Further characterization of charge trapping and de-trapping processes has been realized for investigating the recombination characteristics in OLEDs and OPV devices^45^. Because the EL measurement requires the recombination of holes and electrons, the transportation of the carriers is critical for determining the mobility, especially in unipolar materials which typically exhibits an imbalanced hole and electron transport, and therefore, prohibits from obtaining the information about hole and electron simultaneously. Among the techniques of mobility determination, the TOF measurement estimates the transient signal from the photo-excited carriers. Both the hole and electron mobility can be measured depending on the polarity of the applied voltage^46^. The drawback of the TOF measurement is the requirement for a thick sample (typically several micrometers) for preventing from inaccuracy caused by an insufficient absorption to the excitation source for most materials^47^,^48^,^49^,^50^. Limited selections of commercially available laser sources also restrict the applications of TOF^37^,^47^,^51^,^52^.

Despite these deficiencies, it is still favorable to use TOF measurement in observing the effects of the molecular structures, trap states, scattering centers, and dispersivity on the carrier transport^47^,^53^,^54^,^55^,^56^,^57^,^58^. To overcome these deficiencies, an insertion of charge-generation layer (CGL) which possesses a high absorption coefficient and an energy level matching with the adjacent organic layer has been proposed for confining the carrier-generation width and improves the transient signal for determining the mobility accurately^24^,^37^,^52^,^53^,^58^,^59^,^60^,^61^,^62^. At an early state of the TOF technique, Chen *et al.* used silicon as substrates for measuring the carrier mobility of EL materials, thus showing that the wide wavelength selection of silicon to Nd:YAG laser can act as an efficient CGL and reduces the required thickness of organic layers to the typical value that used in organic electronic devices^63^. Huge progress in exploiting new charge-generation materials was made by Wong and his group, who developed an ambipolar, large-gap, and high-mobility fluorine-based material as a suitable candidate for the use in CGLs^51^,^54^. Wu and his group employed an ethyl-substituted C9 material terfluorene E3, developed by Wong and his group, for simplifying the sample preparation and determines the carrier mobility of a series of commonly used transporting materials that have yet to be characterized by the TOF^60^. By incorporating a specific wavelength that is transparent to the material under test while strongly absorbed by the CGL in the TOF measurement, a simplified sample preparation enables an in-depth investigation into the physical phenomena of the carrier transport^37^,^59^,^61^,^62^. Up to now, the accuracy of the TOF measurement is still under debate, whereas it can meet an approximate evaluation compared with other techniques^64^,^65^,^66^,^67^,^68^.

As an efficient CGL, high absorption and carrier transport are prerequisite for an increased number of photo-excited carriers that contributes to the transient signal through the material under test. These features are present in the photosensitized organic materials that used in OPV devices. In our previous studies, we have fabricated a series of devices with high performance on the basis of the subphthalocyanine (SubPc) donor and C_70_ acceptor^69^,^70^. Based on the same materials system, Pandey *et al.* demonstrated an improved device fabrication process to enhance the device efficiency^71^, thus showing the potential of the combination of SubPc and C_70_ for the use in CGLs. In the current study, we determined the hole mobility of 0.3-μm N,N′-diphenyl-N,N′-bis(1,1′-biphenyl)-4,4′-diamine (NPB) by using the proposed CGL, which provides a well-confined charge-generation width, thus leading to clear non-dispersive properties under various applied voltages. Although a 1-μm NPB was required for obtaining the non-dispersive transient signal, the electron mobility was determined and in good agreement with previous studies. In addition, we proposed a new concept for designing the TOF sample through simply simulating the power-dissipation profiles in the samples. The proposed CGL was thermal-evaporated and can be deposited underneath or atop the mater under test. Therefore, the proposed technique was applicable to determine the carrier mobility of polymeric materials.

## Results

### Thickness-tunable and high-performance SubPc:C_70_ CGL

**Figure 1:**
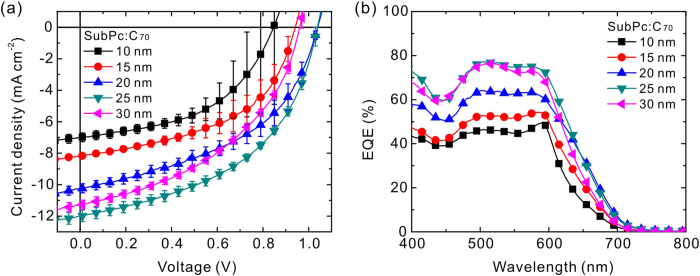
Performance of the OPV devices. **** (**a**) *J*-*V* characteristics for the devices with various SubPc:C_70_ thicknesses in a structure of ITO/SubPc (5 nm)/SubPc:C_70_ (1:5; *x* nm)/C_70_ (43-*x* nm)/BCP (8 nm)/Al (100 nm), *x* = 10, 15, 20, 25, and 30 under AM 1.5G solar illumination at 100 mW cm^−2^. (**b**) Corresponding EQE spectra of these OPV devices.

In the TOF measurement, a thickness-tunable property is preferable without compensation on both the absorption and transport for controlling the charge-generation width because the thickness of a CGL must be considerably thinner than that of the material under test. Therefore, in the current study, OPV devices were fabricated based on a structure of indium-tin-oxide (ITO)/MoO_3_ (15 nm)/SubPc (5 nm)/SubPc:C_70_ (1:5; *x* nm)/C_70_ (43-*x* nm)/bathocuproine (BCP) (8 nm)/Al, where *x* is 10, 15, 20, 25, and 30 nm, for studying the effects of the SubPc:C_70_ thickness on the charge generation and transport properties. The result may be misinterpreted if a single layer of C_70_ was not used because the first optical-field maximum is unlikely to occur within the active layer, as predicted by the optical modeling (Supplementary Fig. S1). Figure 1a shows the current density-voltage (*J*-*V*) characteristics under AM 1.5G illumination at 100 mW cm^−2^ for the devices with various structures. The error bar representing the standard deviation is provided at each data point. The error of fill factor (FF) was lower when the SubPc:C_70_ thickness increased, probably because of the inhomogeneity of the thin film formation at a thinner thickness. Nevertheless, the thickness variation has a minor influence on FF. The difference in open-circuit voltage (*V*_OC_) is probably due to the logarithmic relation between *V*_OC_ and short-circuit current density (*J*_SC_) and the carrier recombination caused by an incomplete interpenetrating network between the SubPc and the C_70_. Such issue, however, is beyond the scope of the current study. The main parameter of special interest here is *J*_SC_, which represents a photon absorption and charge transport properties The parameter *J*_SC_ showed a considerable variation when various SubPc:C_70_ thickness were used. Table 1 summarizes the photovoltaic parameters for all the devices, showing that the best power conversion efficiency (PCE) of 6.0% that can be achieved by a 25-nm SubPc:C_70_. To determine the variation in *J*_SC_, external quantum efficiency (EQE) spectra of the OPV devices were measured, as shown in Fig. 1b. Because *J*_SC_ is the integral of the product of the EQE and AM 1.5G solar spectrum, the averaged EQE in a wavelength range of 500 to 600 nm was directly proportional to *J*_SC_. Although the EQE decreased with the SubPc:C_70_ thickness, these values seem sufficient to allow the carriers for flowing through the material under test in the TOF measurement. These results indicate that the mixture of the SubPc and C_70_ is a promising candidate for the use in CGLs, and its thickness allows room for modulating according to the tested material thickness.

### Applicability of the CGL and the TOF configuration

**Figure 2:**
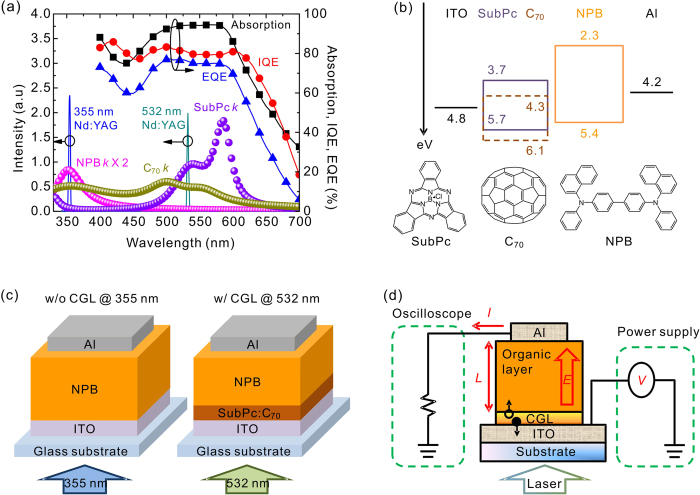
Configuration of the proposed TOF measurement. **** (**a**) Comparison between emission spectra of 355 and 532 nm from Nd:YAG laser and extinction coefficients of organic materials used in the current study. The extinction coefficient of NPB was magnified twofold. Optical properties of OPV device with 6% PCE is provided. (**b**) Energy-level diagram and molecular structures of organic materials. (**c**) Device structures of TOF samples without (w/o) and with (w/) a CGL (SubPc:C_70_) for laser illumination at 355 and 532 nm, respectively. (**d**) Schematic diagram of the TOF measurement with employment of the CGL. The solid and open circles denote electron and hole, respectively. All the structures are not scaled with a real device.

Figure 2a compares the emission spectra at a wavelength of 355 and 532 nm from Nd:YAG laser together with the absorption properties of organic materials used in the current study. As shown in the figure, the NPB has absorption band in a range of 330 to 370 nm, while it is transparent to a wavelength of 532 nm. This explains that the typical wavelength of excitation source is 337 nm (from N_2_ laser) to induce the photogenerated carriers for measuring the carrier transient signal of the NPB without using a CGL^63^,^72^. As a matter of fact, most of organic semiconductors absorb the ultraviolet (UV) light range because of their molecular nature, and therefore previous studies measured the carrier mobility by using 337 or 355 nm to be the excitation wavelength.^60^,^73^,^74^,^75^,^76^,^77^,^78^,^79^,^80^ Some of organic materials, however, especially materials with a wide band gap that having either a low absorption coefficient or an absorption wavelength shorter than the deep UV light, face difficulties in selecting the excitation source and render the inaccuracy in determining the carrier mobility.^60^,^73^,^75^,^76^,^77^ Therefore, a wavelength that is transparent to the wide-bandgap materials while having a high photo-response to the CGL should be a more suitable excitation source. Because the SubPc exhibits wide absorption band from 500 to 600 nm with a shoulder around 532 nm, it is expected that the mixture of the SubPc and C_70_ would be a promising CGL upon the incident wavelength of 532 nm, as the photo-response and charge-generation ability of the OPV device composed of the SubPc and C_70_ was extraordinarily prominent according to the high absorption, EQE, and internal quantum efficiency (IQE) (see Supplementary Figure S2 for detail in determining absorption and IQE). Besides concerning the above properties, the energy level alignment between a CGL and the material under test is crucial for the injection of the photogenerated carriers into the tested material. Figure 2b shows an energy level diagram of TOF sample using the NPB as the tested material and with a SubPc:C_70_ mixed layer as the CGL. The molecular structures of organic materials are provided. The photogenerated holes were easily injected into the NPB because of the absence of energetic barrier between the highest occupied molecular orbital (HOMO) levels at the SubPc:C_70_/NPB interface. However, the lowest unoccupied molecular orbital (LUMO) levels between the CGL and the NPB were not matched, thus resulting in large injection barrier that may impede the electron injection across the SubPc:C_70_/NPB interface. This issue may be resolved through inserting an injection layer and/or applying a relatively high bias to promote the electron injection from the CGL into the NPB, the latter one has been proven to be feasible and thereby the electron mobility was determined as shown in the previous study^72^. Two kinds of experiments were performed in the current study; one is the TOF sample without the CGL under illumination of 355 nm, another incorporates with the CGL under illumination of 532 nm, as shown in Fig. 2c. The laser pulse impinged through the ITO electrode for all the samples. Figure 2d illustrates the setup and working mechanism of the TOF measurement with a CGL using a thickness relatively less than the tested material. The laser-pumped and dissociated carriers were transported through the material with a thickness of *L* under an applied voltage (*V*) from a power supply, which creates an electric field (*E*) to drive holes or electrons, depending on the polarity of the bias applied to ITO. Al electrode collected the carriers, which contribute to the current (*I*) that being recorded by an oscilloscope with a resistor for observing the photovoltage transient signal. By reading the transit time (*t*_T_) from the transient signal, the carrier mobility can be determined using the following equation:




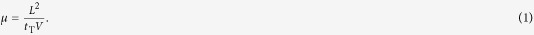




**Figure 3:**
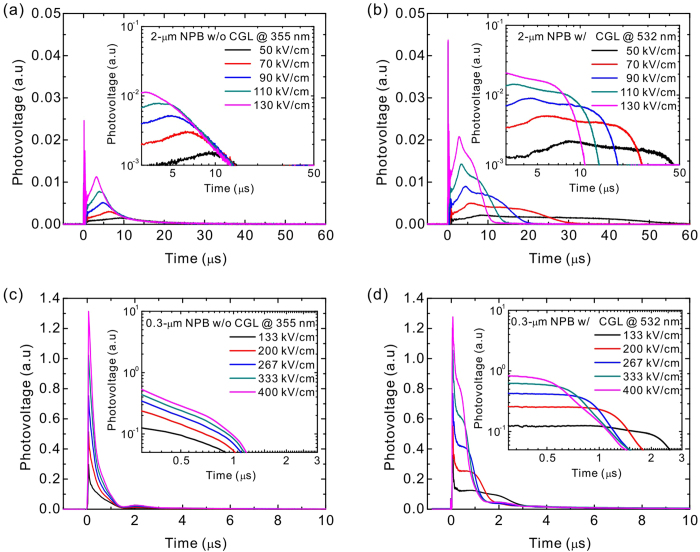
Hole transient signals. **** Hole transient signals for a 2-μm NPB device measured (**a**) without a CGL at a 355-nm excitation and (**b**) with a 100-nm CGL at a 532-nm excitation when various electric fields were applied. Hole transient signals at various electric fields for a 0.3-μm NPB devices measured (**c**) without a CGL at a 355-nm excitation and (**d**) with a 10-nm CGL at a 532-nm excitation. All insets show corresponding log-log plots. The terms w/o and w/ represent the without and with, respectively.

**Comparisons of the hole transient signals for the 2- and 0.3-μm NPB without and with the CGL.** Under the photoexcitation, excitons were dissociated within the SubPc:C_70_ CGL and generates holes and electrons that are transported by SubPc and C_70_ molecules, respectively. To measure the hole transient signal, a positive bias was applied to the ITO electrode, photogenerated holes were swept through the NPB layer and reaches the Al electrode. Figure 3a shows the hole transient signal for the structure of ITO/NPB (2 μm)/Al (100 nm) at various electric fields under 355-nm illumination, together with an inset showing a corresponding log-log plot. The NPB is a non-dispersive material, which showed a clear plateau and dramastic drop at the turning point in the transient signal^57^,^58^. In our case, however, the emergence of cusps were observed, especially in high electric fields. The presence of cusps has been observed in previous studies, which have attributed this phenomenon to the intrinsic feature between the randomly disordered transport sites which act as trap states for the carriers, thus leading to the monotonically increased current instead of forming a plateau^37^,^49^,^62^,^79^,^81^. Here, the NPB thickness of 2 μm only may also lead to a different results from previsous studies that used the NPB thickness of 7-10 μm. Although the reason for the emergence of the cusps is not well understood, the transient signal plotted in a log-log scale enabled determining the transit time from the intersections of the asymptotes to the increasing signals and tail sections, occasionally coincides with the cusps in the linear plot. By contrast, the sample configuration of ITO/CGL (100 nm)/NPB (2 μm)/Al (100 nm) under 532-nm excitation showed very different transient signals, as shown in Fig. 3b. The cusps were still observed, whereas the tail sections drop more rapidly at different applied electric fields, as observed in the log-log plot. This observation is ascribed to the well-confined position of the charge generation by using the CGL with a thickness relatively less than that of the NPB layer. In addition, the NPB is transparent to a wavelength of 532 nm, and therefore only the thin CGL can generate the photoexcited carriers and reaches the Al electrode simultaneously because of the non-dispersive characteristic of the NPB. Therefore, a clear turning point between the plateau and the tail section well defineed the transit time either in a linear or log-log plot, when the degree of dispersion was reduced. In order to determine the dispersion property, we used the general expression describing the dispersivity as follows^55^,^82^,^83^:




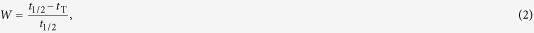




where *t*_1/2_ is the time that the photovoltage drops to the half of the value of the transit time (*t*_T_). A lower *W* indicates a less spread of the carrier transport through materials, thus leading to an abrupt drop in the tail section instead of a slow descent. In general, the main idea of estimating the *W* parameter is for observing the effects of traps on molecularly doped materials because carrier spread was brought about by these trap states that may dominate the dispersivity of materials^55^,^82^,^83^. However, the carrier spread during the transport can be influenced by the photogenerated carrier profile, because the distance that required for reaching the Al electrode for the carriers generated at different positions cannot approximate to be constant when the charge-generation width is wide and comparable to the thickness of the material under test. Therefore, we introduce the dispersion parameter to investigate the dispersivity of the tested TOF samples. Table 2 summarizes the dispersion parameters for all the samples (Supplementary Figure S3), except for the 0.3-μm NPB without the CGL because the transient time was indistinguishable in this sample. At an incident wavelength of 355 nm for the 2-μm NPB without the CGL, the values of *W* was in a range of 0.25-0.30. This value on average is twofold higher than that reported in previous studies that obtained the value of *W* to 0.13 for neat NPB^82^. We attributed this substantial difference to the relative thin NPB thickness of 2 μm only in the current study compared with the typical values, 7-10 μm, in previous studis^57^,^58^,^82^. When a 10-nm CGL and 532-nm excitation source were used, the value of *W* decreased to less than 0.10 on average, which was lower than the value reported previously, thus indicating a more non-dispersive property. This result was attributed to the well-confined charge-generation width as will be discussed in detail later. However, when the thickness of the NPB decreased to 0.3 μm, the transient signal was dispersive and the transit time is indeterminable without using the CGL, as shown in Fig. 3c. This was mainly caused by the insufficiently high absorption coefficient of the NPB at 355 nm and thereby resulting in a broad charge-generation region. By contrast, when the 0.3-μm NPB and the CGL was used, the plateaus followed by the tail sections were cleearly observed in the transient signals at various applied electric fields under excitation of 532 nm, thus permitting the determination of the transit times and the carrier mobility, as shown in Fig. 3d. When the thickness of the NPB reduced from 2 to 0.3 μm, the extracted *W* values slightly increaseed from 0.07 to 0.12 on average, which is similar to the value obtained from the NPB with the thickness of 7-10 μm under an excitation of 337 nm. Because of the different absorption properties between the CGL and the material under test (i.e., the use of an excitation of 532 nm at which the SubPc:C_70_ has a high absorption while is transparent to the NPB), we reduced the TOF sample thickness to 0.3 μm, which is the thinnest value for determining the hole mobility of the NPB to date.

### Observing the charge-genaration region by the power-dissipation profiles in the TOF samples

**Figure 4:**
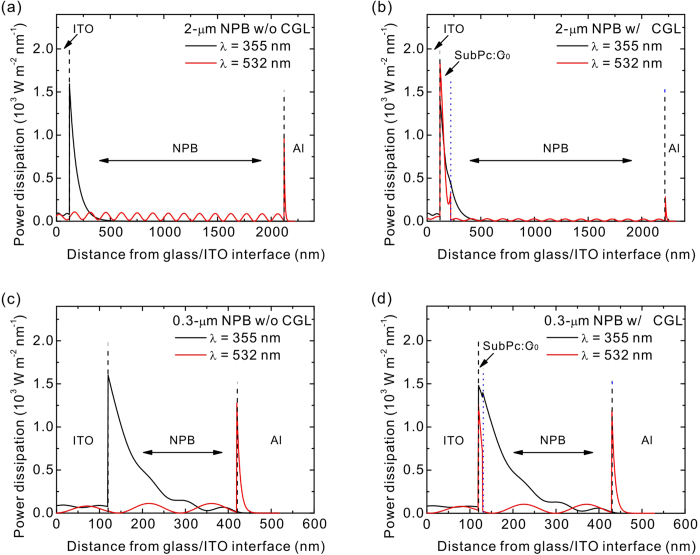
Power-dissipation profiles inside the TOF samples. **** Power-dissipation profiles in TOF samples at illumination of 355 (black lines) and 532 nm (red lines) for a 2-μm NPB (**a**) without and (**b**) with a 100-nm CGL, and 0.3-μm NPB (**c**) without and (**d**) with a 10-nm CGL. The terms w/o and w/ represent the without and with, respectively. The region of the CGL is indicated by arrows.

To determine the reduced degree of dispersion when the CGL was used, we proposed a new approach to characterize the generation width of photoexcited carriers that is crucial for contributing to the dispersivity of the transient signal. On the basis of the transfer matrix method as proposed by Pettersson *et al.*, power-dissipation profiles enabled predicting optical-field distribution and power dissipation in thin-film based devices^84^. Using the same optical constants of the organic materials that were used in predicting the optical conditions of OPV devices (Supplementary Figure S1), the power-dissipation profiles in the TOF samples with different structures were calculated to investigate the dispersive issues arising from both the sample thickness and wavelength selection. Figure 4a shows the power-dissipation profiles as a function of layer thickness in the TOF samples composed of the 2-μm NPB without the CGL at 355- and 532-nm wavelengths. Because of the transparency of the NPB to 532 nm, no photocarriers can be generated. For a wavelength of 355 nm at which the NPB features an absorption peak, the photo-excited carriers were populated near the ITO electrode and the carrier density decays exponentially when the film thickness increased. This prediction showed a broad charge-generation region in a range of approximately 200 nm. Therefore, the carriers generated at a different spatial position may contribute a dispersive transient signal, as shown in Fig. 3a, because the carriers cannot reach the Al electrode simultaneously. Therefore, the thickness for determining the transit time accurately of the NPB requires a thicker layer over ten times higher than the charge-generation region, which explains why TOF samples typically have a thickness over several micrometers^57^,^58^,^72^,^82^, in particular the material under test which has a low absorption coefficient, thus resulting in a broad charge-generation region^24^,^47^,^48^,^49^,^50^,^53^,^55^,^68^. By contrast, employing the CGL formed by the SubPc and C_70_ mixture which absorbs an incident wavelength of 532 nm and provides a well-confined charge-generation width of less than 100 nm, as shown in Fig. 4b, achieving more non-dispersive transient signals because the photovoltage drops abruptly as the photogenerated carriers arrive at the Al electrode without dispersion (see Fig. 3b). This is a remarkable finding because typical CGLs based on small-molecular materials have similar absorption properties such as a low absorption coefficient and/or overlapped absorption band with respect to the material under test. Therefore, the possibility of exciting both the CGL and tested material with the same excitation wavelength may occur, as shown in Fig. 4b. An unexpected charge-generation region was observed, and therefore, a thick sample thickness is required in previous studies^53^,^58^,^60^,^82^. When the NPB thickness decreased to 0.3 μm, the charge-generation region almost covered the entire film thickness (Fig. 4c), and therefore the transit time is impossible to determine, as shown in Fig. 3c. When a thin-layer CGL with 10 nm was used in a 0.3-μm NPB, the charge-generation width was clearly defined within the CGL under illumination of a 532-nm excitation, as shown in Fig. 4d. The carriers reached the Al electrode without dispersion and the transient signal was dominated by the distribution of density of states in the NPB, as shown in Fig. 3d. These results suggested two issues in the TOF measurement. One is that the thickness of materials under test do affect the determination of the transient time and dispersivity. Secondly, the selection of excitation source with regard to the CGL and tested material can greatly impact on the degree of dispersion because the charge-generation profile can be varied at different wavelengths according to the absorption of materials under test.

### Thickness-dependent hole and electron mobility

**Figure 5:**
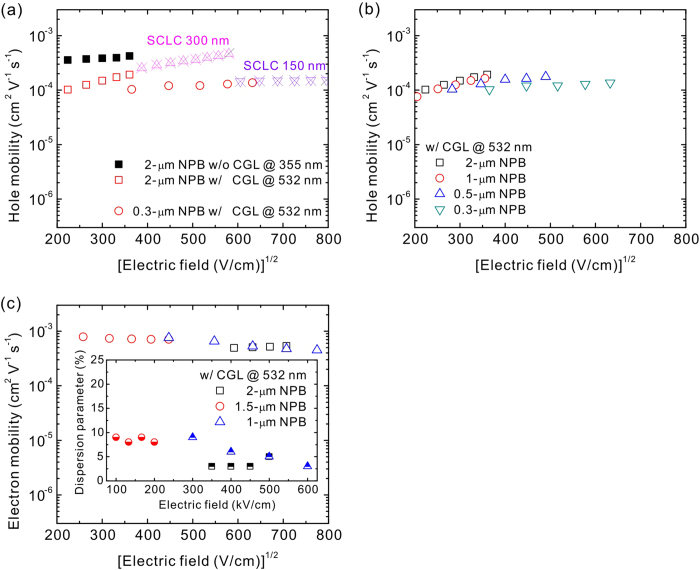
Thickness and electric-field dependence of carrier mobility. **** (**a**) Hole mobility measured with different configurations in the current study compared with the values obtained by the SCLC model on the basis of hole-only devices. (**b**) Hole mobility at various NPB thicknesses measured using the TOF measurement with a CGL. (**c**) Electron mobility at various NPB thicknesses measured using the TOF measurement with a CGL. The inset shows electron dispersion parameters as a function of applied electric fields for the TOF samples with various thicknesses of the NPB. The terms w/o and w/ represent the without and with, respectively.

Figure 5a shows the hole mobility estimated at various film thicknesses and excitation sources. For comparison, the mobility obtained from a hole-only device, in a structure of ITO/MoO_3_ (15 nm)/NPB (150 or 300 nm)/Au, detertemed using a SCLC model is provided (Supplementary Figure S4)^41^,^42^. The 0.3-μm NPB without the CGL cannot determine the hole mobility because of the lack of a turning point in the transient signals, as shown in Fig. 3c. The 2-μm NPB without using the CGL at 355-nm excitation has a compable hole mobility of 2-4 x 10^−4^ cm^2^ V^−1^ s^−1^ to previous studies^57^,^58^,^63^,^82^. When the CGL and 532-nm excitation were used in the 2-μm NPB device, the hole mobility becomes lower in a range of 1-2 x 10^−4 ^cm^2^ V^−1^ s^−1^ and exhibited high dependence on the electric field. We attribute this phenomenon to the confinement effect of the charge generation because of the use of the CGL, thus leading to a longer distance the carriers require to travel in comparison to the carriers generated with a broad carrier distribution in the samples without the CGL. As a matter of fact, the carriers generated in the broad distribution may overestimate the carrier mobility because the averaged travelling distance is shorter than the case if the carriers are generated in a sheet. The 2-μm NPB sample used the 100-nm CGL, which is one-twentieth of the 2-μm NPB, for generating the carriers. The CGL was the SubPc:C_70_ mixture, which may hinder the carrier transport at such thick layer because of the incomplete interpenetrating network and/or short carrier lifetime. Therefore, a CGL possessed a better carrier transport is expected to obtain more reliable data. This can be achieved by reducing the thickness of the CGL to increase the thickness ratio of NPB to CGL. In the TOF sample composed of a 0.3-μm NPB with a 532-nm excitation, the carrier mobility remains almost the same of approximately 1 x 10^−4^ cm^2^ V^−1^ s^−1^ with a slight dependence on electric field. Because the CGL was reduced to 10 nm which is one-thirtieth of the NPB layer, the photogenerated carriers were appropriately-confined in a narrow width and can transport through the NPB layer without being trapped in the SubPc:C_70_ mixture. Although the hole mobility obtained based on the use of the CGL differed between the devices with varous thicknesses, the values were on the order of 10^−4^ cm^2^ V^−1^ s^−1^ (Fig. 5b), thus showing a thickness-iudependent property. By contrast, the carrier mobility estimated by the SCLC model lead to a substantial thickness-to-thickness variation, as observed in the current study and in previous studies^41^,^42^,^65^. Therefore, the TOF measurement with the employment of the CGL and specific excitation source is more reliable and becomes a valuable tool in determining the carrier mobility once the sample thickness can be reduced to a value near that used in a real device. The hole mobility was determined because of an appropirate energy-level alignment between the CGL and the NPB. However, the electron mobility was yet to determine becuaae large barrier height for electron at the CGL/NPB interface may impede the electron injection (Fig. 2b) and limits the proposed technique to estimating the hole transport only. To address this issue, we determined the electron mobility by applying a negative bias to ITO, thus allowing electrons to be swept toward the Al electrode. The samples with the 10-nm CGL at 532-nm excitation showed non-dispersive properties in a wide range of the NPB thicknesses from 2 to 1 μm (Supplementary Figure S5). The electron transient signals showed clear turning points differentiated from the plateaus and tail sections, defining the transit times without ambiguities (Supplementary Table S1). Figure 5c shows the electron mobility estimated from these transit signals, together with an inset presenting the dispersion parameter as defined in Equation 2. The electron mobility measured in the current study was 5-8 x 10^−4^ cm^2^ V^−1^ s^−1^, which agrees well with the value reported by Tse *et al.*^72^, thus proving that the NPB is an ambipolar material with a higher electron mobility than the hole moblity. In addition, the dispersion parameters for electron were lower than 0.1 for all thicknesses and electric fields, indicating the highly non-dispersive electron transport of the NPB. We inferred that the high barrier for electrons at the CGL/NPB interface could be overcome by high electric fields that promotes electron injection, thus permitting the measure of electron transport in the NPB. Although the thickness for determining electron mobility was limited to 1 μm, this thickness is the thinnest value to date. We have successfully demonstrated and re-evaluated that the TOF technique is a valuable and practical tool in determining carrier mobility using a combination of the highly efficient OPV materials SubPc and C_70_. In the near future, carrier-injection layers or structural designs are required to reduce the TOF sample thickness to achieve the real-device thickness.

### Flexibility of the proposed CGL

**Figure 6:**
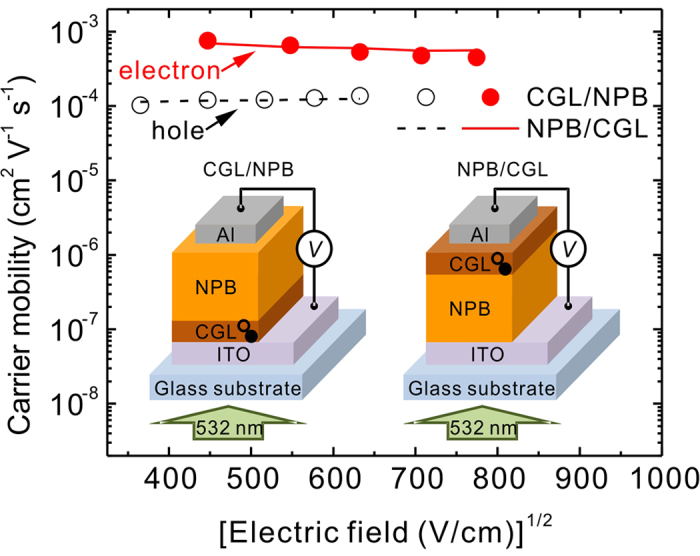
Comparison of CGLs deposited underneath and atop the NPB. **** Hole and electron mobility obtained from the various device structures with depositing CGLs underneath and atop NPB layer. The open circles and dashed lines indicate the hole mobility of the NPB. The solid circles and lines indicate the electron mobility of the NPB. The inset depicts CGL/NPB and NPB/CGL structures. In the inset, the open and solid symbol denote the hole and electron, respectively.

The discussed devices were placed the proposed CGL underneath the material under test. This approach may limit to the samples prepared by thermal evaporation. For example, polymers are mostly spun-coated onto substrates and thus may dissolve CGLs that are already deposited on the substrates. Therefore, we proposed that another advantage of using the proposed CGL is the flexibility of the fabrication process (i.e., the thermal-evaporated CGL). For measuring the carrier mobility of polymers, the discussed structure can be altered to ITO/polymer/CGL/Al to resolve the dissolving problem. To demonstrate this idea, a structure of ITO/NPB (0.3 μm)/CGL (10 nm)/Al was fabricated for comparison with ITO/CGL (10 nm)/NPB (0.3 μm)/Al. Because the CGL is on the top of the NPB, the measurement setup is different from the case in which a CGL is underneath the NPB (Supplementary Figure S6). Figure 6 compares the results obtained from both the CGL/NPB and NPB/CGL structures. The hole and electron mobility were mostly identical for both device structures, thus indicating that in the thermal-evaporation system the CGL can place underneath and atop materials of interest, both of which showed an accurate measure of the carrier mobility. Therefore, for measuring the carrier mobility of polymers, the proposed CGL can be deposited on polymers that were already spun-coated onto the substrates without concerning the dissolving problem. To demonstrate the possibility of confining the charge-generation width by using the proposed CGL for measuring the carrier mobility of polymers that were used in OPV devices, we choose three commonly used polymers, poly[(R)-3-(4-(4-ethyl-2-oxazolin-2-yl)phenyl)thiophene] (PEOPT)^85^, poly(3-hexylthiophene-2,5-diyl) (P3HT)^86^, and [6,6]-phenyl-C61-butyric acid methyl ester (PCBM)^87^ as a standard reference. The structure of TOF sample was ITO/polymers/CGL/Ag, in which the Ag must be transparent (the thickness is typically 10-20 nm) for the light passing through the Ag to excite CGLs^88^. The calculated power-dissipation profiles were shown in the Figure S7 in the Supplementary Information. The measurement configuration for measuring the polymers are provided (Supplementary Information Figure S7). To simulate a real case, the thicknesses of the polymers were fixed at 100 nm and that of the CGL was fixed at 5 nm. Because PEOPT and P3HT absorb the wavelength of 532 nm, exciting the CGL with a 355-nm excitation is preferable. The transient signals may be primarily contributed by the carriers in the CGL because the power dissipation in the CGL was much higher than that in the polymers. However, for the PCBM, which has a high absorption in a wavelength of 355 nm, a 532-nm excitation will be more appropriate to excite the CGL and the transient signals were primarily contributed by the carriers in the CGL. Although the charge generation may occur in the polymers, most of the carriers were generated in the CGL and contributes to the transient signals, thus leading to a more accurate carrier mobility. In the state of the art, a series of novel polymers was synthesized toward the near-infrared (NIR) absorption for extending the spectral coverage over the solar spectrum^18^,^89^,^90^,^91^,^92^,^93^,^94^. The polymers were the so called low-bandgap polymers which having absorption within an NIR wavelength range. In addition, the proposed CGL has high extinction coefficient than that of these polymers, especially when these polymers were in a blend with the PCBM. Although the proposed CGL cannot completely confine the charge-generation width in the polymers, the CGL can contribute most of the carriers to dominate the transient signals without overestimating the carrier mobility of the polymers because of a broad width of charge generation.

## Discussion

The TOF measurement is widely used to evaluate the carrier mobility of organic materials over the last decade. To obtain a plateau and an abrupt drop of the transient signal, the sample thicknesses were typically several micrometers. However, in practical electronic devices the thickness is between tens to hundreds nm, or sub-micrometers. The requirement of a thick TOF sample is primary due to an unwell-confined charge-generation width. This requirement could have resulted in overestimating carrier mobility because the travelling distance of carriers is not equivalent to the thickness of materials under test, especially parts of carriers travel a shorter distance. Although the concept and the use of a CGL for confining the charge-generation width is not new, concerning that both small-molecule and polymeric CGLs have been reported^24^,^59^,^60^, the proposed CGL is advantageous over those reported CGLs. For example, the reported small-molecule CGLs have a wide band gap and thereby absorb a wavelength of 355 nm substantially. However, the high energy of the 355-nm wavelength may excite the excitons in the materials under test simultaneously, thus rendering the estimation of the carrier mobility inaccurate. The proposed CGL has considerably high quantum efficiency, such as absorption, IQE, and EQE, in a wavelength range of 500 to 600 nm. The excitation source can be changed from 355 to 532 nm, both of which are unlikely to be absorbed by most materials that were used in OLEDs. To measure materials that were used in OPV devices, the proposed optical-simulation approach is valuable for estimating the charge-generation profile in devices under test. Therefore, the charge-generation width can be well controlled for obtaining a non-dispersive transient signal and determines the carrier mobility accurately. In addition, the proposed CGL composed of a blend of a donor and an acceptor on the basis of a bulk heterojunction. Therefore, both holes and electrons can be generated to materials under test for measuring the hole and electron mobility. These results demonstrated the applicability of using OPV materials to be an effective CGL in the TOF measurement. Furthermore, these results also presented a new application of OPV devices in addition to light harvesting.

In the current study, the TOF samples with two NPB thicknesses were compared. To avoid the unnecessary voltage drop across the CGL, the thickness ratios of the NPB to the CGL were 20:1 and 30:1 for the 2-μm and 0.3-μm NPB, respectively. However, the ratios were not necessarily a reflection of reducing the CGL thickness when a thinner NPB thickness was used. To address this issue, the CGL thicknesses of 5, 10, and 15 nm were used in the TOF samples with a 0.3-μm NPB. The transient signals of the samples under 532-nm illumination showed almost unchanged profiles and identical hole mobility (Supplementary Figure S8). This result indicates that the 5-nm CGL enabled measuring a thinner thickness of the TOF samples. In previous studies, some unexpected doping effect such as oxygen or intrinsic dispersivity may negatively affect the transient signal, resulting in a dispersive transient signal and cannot determine the transient time accurately. The advantage of using CGLs was for confining the charge-generation width to determine the transient time accurately. Therefore, the proposed approach of using the OPV CGL may not be a solution to overcome this doping issue. A possibility to resolve this problem is to directly remove the impurities which form defects and influence the carrier transport through the purification of materials under test (Supplementary Figure S9). In addition, the aforementioned result suggested that the proposed CGL is applicable for determining the carrier mobility of polymers. To demonstrate the possibility, a P3HT-based TOF sample with a 5-nm CGL was fabricated and tested. The different illuminating direction was also conducted for proving the accuracy. Although the P3HT/SubPc:C_70_ unintentionally gave rise to a P3HT/C_70_ bilayer OPV structure, the hole mobility showed consistent results independent of the illuminating direction (Supplementary Figure S10).

In summary, we used OPV materials, SubPc and C_70_, that having a high PCE of up to 6% in an OPV configuration, to be an efficient CGL for the TOF measurement. By comparing the NPB-based TOF samples without and with the CGL under 355- and 532-nm photoexcitation, respectively, the transient signals at various applied electric fields become more non-dispersive, and therefore the transit time can be determined accurately, as reflected by the reduced degree of dispersion parameter. In the observations of the power-dissipation profiles in the TOF samples, the reduced dispersivity was attributed to the use of a specific excition wavelength of 532 nm which is transparent to the NPB while highly absorbed by the CGL, thus permitting a well-defined width for charge generation. Combined with the use of the CGL and the selected excitation source, the thickness of the TOF samples for determining the hole and electron mobility of the NPB can be reduced down to 0.3 and 1 μm, respectively, both of which are the thinnest sample thickness reported to date. Our findings provided not only a novel design rule to prepare the TOF samples with a thickness approaching the thickness that was used in a real device, but also presented a prospective use of photovoltaic materials to be an efficient CGL for obtaining a more reliable data for the TOF measurement in determining the carrier behavior of typical transporting materials with a wide band gap. In addition, the fabrication flexibility of the proposed CGL (i.e., the thermal-evaporated process enables fabricating the CGL underneath or atop the material under test) was not limited to measure the carrier mobility of small molecules but also polymers by simply changing the sample preparation method.

## Methods

### Materials and Device Fabrications

All materials, MoO_3_, SubPc, C_70_, BCP, NPB, Al were purchased from Sigma-Aldrich. The SubPc was purified twice prior to use, whereas the rest of the organic materials were used as received. ITO-coated glass substrates were cleaned with ultrasonic bath in solutions (detergent, deionized water, acetone, isopropanol) without further surface treatment, either for OPV devices or TOF samples. The thin film deposition was conducted in a high-vacuum chamber (<8 × 10^−6^ Torr). The deposition rates and film thicknesses were monitored during the deposition using a deposition controller (Sycon Instruments XTM-2XM) with a quartz crystal microbalance. The film thickness of each layer was measured using a surface profiler (Veeco Tektak 3) and an ellipsometry (Raditech SE-950). The deposition rate of each layer in the OPV devices was controlled at approximately 0.1 nm/s. To form a SubPc:C_70_ mixture in a volume ratio of 1:5, the deposition rates of the SubPc and C_70_ were independently controlled to approximately 0.04 and 0.20 nm/s, respectively. For the TOF samples, NPB layers were deposited at 0.3-0.5 nm/s, for both 2 and 0.3 μm. The deposition of Al cathode through a shadow mask defined the active area of 0.04 cm^2^ for both the OPV devices and TOF samples. After the cathode deposition, the devices were appropriately encapsulated using an UV-curable epoxy resin (Everwide Chemical Co. Ltd. EXC345) and a getter-attached cover glass for preventing the organic layers from the ambient environment. A home-made *in*-*situ* and independently controlled shutter system in which each shutter can be individually controlled was used to fabricate the devices with various structures in one run without breaking the vacuum, thus preventing the deviation between the production runs. A home-designed layout on the ITO substrates enabled producing five devices on one substrate. A total number of fifteen devices were fabricated simultaneously on three different substrates.

### OPV Devices Characterizations

All characterizations were performed in the air. *J*-*V* characteristics were measured using a source meter (Keithley 2636A). Photo *J*-*V* characteristics were performed under AM 1.5G solar illumination (Newport 91160A) at 100 mW cm^−2^ which was measured using a silicon reference cell (PV measurement; area: 3.981 cm^2^). The EQE spectra were determined using a monochromator (Newport 74100) for generating monochromatic light in a wavelength range of 400 to 800 nm and a lock-in amplifier (Stanford Research Systems SR830) chopped at 250 Hz for recording the photocurrent from the OPV devices.

### Materials Characterizations

HOMO levels and work functions of thin films were evaluated from high-energy onset points in a spectrum measured using a photoelectron spectrometer (Riken Keiki AC-2). Energy gaps were derived from the absorption spectra by the high-wavelength onset points recorded using a UV-visible spectrophotometer (Thermo Scientific Evolution 220) for estimating LUMO levels of the organic materials. Optical constants, refractive indices and extinction coefficients, were determined using the ellipsometry that was used in the measurement of film thickness. EL spectra of laser emission were measured using a spectrometer (Ocean Optics USB2000+).

### TOF Measurements

An Nd:YAG pulsed laser (Spectra-Physics Quanta-Ray INDI-40-10) with second and third harmonic wavelengths, 355 and 532 nm, respectively, were used as excitation sources. The pulse width was in a range of 6-9 ns. The power of 355- and 532-nm laser was approximately 20-30 mW measured using a power meter (Scientech AC2501). The laser beam diameter was approximately 10 mm, which covered the entire area of the TOF samples. The source meter that was used in characterizing OPV devices provided the electric field across the TOF samples for driving photo-excited carriers. The transient signals were recorded using a 2.5 GHz oscilloscope (LeCroy WaveRunner 625Zi) triggered synchronously with the Q-switch Nd:YAG laser. For the NPB TOF samples, all excitation illuminated through the ITO side. For polymer TOF samples, the excitation illuminated from both ITO and Ag sides.

## Author Contributions

S.-W.L. and C.-C.L. contributed equally to this work. S.-W.L. and C.-C.L. provided the idea of this research and wrote this paper. W.-C.S. and C.-H.Y. measured the TOF samples and analyzed the data. C.-F.L., K.-T.C. and Y.-S.S. fabricated the TOF samples. Y.-Z.L., T.-H.S. and Y.-H.L. summarized the references and provide the information about writing this paper. B.-Y.H. and W.-C.C. developed the high-performance OPV devices.

## Additional Information

**How to cite this article**: Liu, S.-W. *et al.* Downscaling the Sample Thickness to Sub-Micrometers by Employing Organic Photovoltaic Materials as a Charge-Generation Layer in the Time-of-Flight Measurement. *Sci. Rep.* **5**, 10384; doi: 10.1038/srep10384 (2015).

## Supplementary Material

Supplementary Information

## Figures and Tables

**Table 1:** Performance of the OPV devices.

Device	*V*_OC_ (V)	*J*_SC_ (mA cm^−2^)	FF (%)	PCE (%)
10 nm	0.85 ± 0.11	7.0 ± 0.3	49 ±3	2.9 ± 0.5
15 nm	0.94 ± 0.03	8.2 ± 0.1	50 ±4	3.9 ± 0.4
20 nm	1.04 ± 0.01	10.2± 0.3	47± 3	5.0 ± 0.2
25 nm	1.04 ± 0.01	12.0± 0.4	48 ±1	6.0 ± 0.2
30 nm	0.96 ± 0.01	11.3± 0.3	45 ±1	4.9 ± 0.1

Photovoltaic parameters of OPV devices obtained under AM 1.5G solar illumination at 100 mW cm^−2^. The standard deviation for an average of fifteen devices are provided.

**Table 2:** Transient time and dispersivity.

**2-μm NPB w/o CGL @ 355 nm**				**2-μm NPB w/ CGL @ 532 nm**				**0.3-μm NPB w/ CGL @ 532 nm**			
**E**	***t***_**T**_ **(μs)**	***t***_**1/2**_ **(μs)**	**W**	**E**	***t***_**T**_ **(μs)**	***t***_**1/2**_ **(μs)**	**W**	**E**	***t***_**T**_ **(μs)**	***t***_**1/2**_ **(μs)**	**W**
50 kV/cm	11.2	–	–	50 kV/cm	39.2	–	–	133 kV/cm	2.19	2.48	0.12
70 kV/cm	7.68	10.4	0.26	70 kV/cm	22.9	25.1	0.09	200 kV/cm	1.25	1.41	0.11
90 kV/cm	5.78	7.71	0.25	90 kV/cm	14.9	15.7	0.05	267 kV/cm	0.93	1.04	0.11
110 kV/cm	4.63	6.47	0.28	110 kV/cm	10.5	11.2	0.06	333 kV/cm	0.70	0.82	0.15
130 kV/cm	3.65	5.22	0.30	130 kV/cm	8.01	8.55	0.06	400 kV/cm	0.55	0.68	0.19

Hole transit times and dispersion parameters at various applied electric fields for the TOF measurements in this study. The terms w/o and w/ represent the without and with, respectively.
