# Supplementary material for: Downscaling the Sample Thickness to Sub-Micrometers by Employing Organic Photovoltaic Materials as a Charge-Generation Layer in the Time-of-Flight Measurement
Source: Sci Rep. 2015 May 22;5:10384. doi: 10.1038/srep10384 (PMC4441200; doi:10.1038/srep10384)
Supplement: Supplementary Information [file srep10384-s1.doc]

Supplementary Information

Downscaling the Sample Thickness to Sub-Micrometers by Employing Organic Photovoltaic Materials as a Charge-Generation Layer in the Time-of-Flight Measurement

Shun-Wei Liu^1^,*, Chih-Chien Lee^2^,*, Wei-Cheng Su^2^, Chih-Hsien Yuan^2^, Chun-Feng Lin^1^, Kuan-Ting Chen^2^, Yi-Sheng Shu^1^, Ya-Ze Li^1^, Tsung-Hao Su^1^, Bo-Yao Huang^2^, and Wen-Chang Chang^2^ & Yu-Hsuan Liu^2^

Correspondence and requests for materials should be addressed to: S.-W. Liu ([swliu@mail.mcut.edu.tw](mailto:swliu@mail.mcut.edu.tw)) and C.-C. Lee ([cclee@mail.ntust.edu.tw](mailto:cclee@mail.ntust.edu.tw))

^1^Department of Electronic Engineering, Ming Chi University of Technology, New Taipei City 24301, Taiwan

^2^Department of Electronic Engineering, National Taiwan University of Science and Technology, Taipei 10607, Taiwan

**1. Optical simulation on the energy dissipation profiles for the OPV devices**

On the basis of the general multilayer structure of OPV devices, the transfer matrix method as proposed by Petterson *et al.* enables to the prediction of the optical-field distribution and energy dissipation inside the OPV devices once the optical constants, refractive index and extinction coefficient, are determined and the device configuration is given.^[1]^ By assuming the incident light propagates perpendicular to the substrate and the optical constants are linearly concentration-dependent, the optical-field distribution can be calculated accordingly and the energy dissipation *Q*(*z, λ*) at position *z* under incident wavelength of *λ* can be predicted by^[1]^

$Q\left(z,\lambda\right)=\frac{1}{2}c\varepsilon_{0}\alpha n\left|E_{0}\right|^{2}$, (S1)

where *c* is the speed of light, *ε*_0_ is the permittivity of free space, *α* is the absorption coefficient, *n* is the refractive index, and |*E*_0_|^2^ is the normalized optical-field intensity to the incident light intensity. The device structure configured of only a single mixture of SubPc:C_70_ is ITO (120 nm)/SubPc:C_70_ (1:5; 15, 20, 25, and 30 nm)/BCP (8 nm)Al (100 nm). **Figure S1a-d** shows the calculated energy dissipation profiles for these OPV devices. It is obvious that the first optical interference takes place in ITO instead of the active layer, resulting in undesirable optical-field distributions and hence the device photo-characteristics. In contrast, to elucidate the photo-response and transport property we use a planar-mixed heterojunction in a structure of ITO (120 nm)/SubPc (5 nm)/SubPc:C_70_ (1:5; 43-*x* nm)/C_70_ (*x* nm)/BCP (8 nm)Al (100 nm), where *x* is 15, 20, 25, and 30 as a standard device configuration. As shown in **Figure S1e-h**, the active layers cover the whole interference maxima and, more importantly, the mixing layer SubPc:C_70_ has comparable energy dissipation, allowing the observation of the thickness-dependent absorption and transport properties as proposed in the main text.

**Figure S1.** Calculated energy dissipation profiles for the OPV devices (a-d) with a single mixture of SubPc:C_70_ and (e-h) with a planar-mixed heterojunction at different thicknesses.

**2. Estimation of the total absorption and IQE for the OPV devices**

Total absorption measurement was done in an integrating sphere with a reflection mode to take the interference, scattering, and electrode absorption into account in a real OPV device.^[2]^ As shown in **Figure S2a**, a light is incident perpendicularly on the sample and a detector collects the light that is integrated by the sphere. A baffle was used to avoid the light directly impinging on the detector. The reflection mode calculates the ratio of reflected light (*I*_R_) from the sample to initial light intensity (*I*_0_) as shown in **Figure S2b**. The transmission is negligible in a real OPV device because the absorption is primarily dominated by the optical interference due to the use of a reflective electrode. Therefore, the total absorption of the OPV device can be approximated to 1-*I*_R_/*I*_0_. Because EQE is the product of IQE and absorption efficiency, or the total absorption, the IQE is hence estimated by dividing EQE by total absorption.

**Figure S2.** (a) Schematic diagram of the total absorption measurement in an integrating sphere. (b) Illustration of the reflection mode to determine the reflection for the OPV devices.

**3. Determination of the hole transit time and dispersion parameter of the TOF samples**

**Figure S3** shows the replicates of the log-log plots of Figure 3 in the main text. The *t*_T_ are clearly indicated by an arrow together with a text. The dashed lines indicate the *t*_1/2_.

**Figure S3.** Replicates of the log-log plots of Figure 3 in the main text. The intersections of the asymptotes to the plateau (or increasing signal) and the tail section are marked by an arrow together with a text indicating the transit time (*t*_T_). The times required for photovoltage drops to half of its value at the transit time, denoted as *t*_1/2_ in the main text, are marked with vertically dashed lines. The terms w/o and w/ represent the without and with, respectively.

**4. SCLC measurement for the hole-only devices**

Hole-only devices consist of ITO/MoO_3_ (15 nm)/NPB (150 or 300 nm)/Au. The SCLC can be described as^[3]^

$J=\frac{9}{8}\varepsilon\varepsilon_{0}\mu\frac{V^{2}}{L^{3}}$, (1)

where *ε* and *ε*_0_ are the dielectric constant and the permittivity of the free space, *µ* is the carrier mobility, *V* is the applied voltage across the device, and *L* is the thickness of the organic layer. Note that the *ε* of the hole-only device was measured by a precision LCR meter (Agilent E4980A). **Figure S4** shows the experimental and fitting SCLCs, both of which are in good agreement, thus indicating an accurate estimation of the hole mobility of the NPB.

**Figure S4.** SCLCs for the hole-only devices with different NPB thicknesses plotted in a semi-log scale. The inset is the corresponding result in a log-log plot. The open symbols and solid lines are the experimental and fitting results, respectively.

**5. Determination of the electron transient signal of the TOF samples with the CGL**

**Figure S5** shows the electron transient signal for the TOF samples using the CGL with different NPB thicknesses. The thickness ratio of the CGL to the NPB was kept at 1:30. The *t*_T_ are clearly indicated by an arrow and with a text. **Table S1** summarizes the transit times and dispersion parameters for the electron transport.

**Figure S5.** Electron transient signals at various applied electric fields for the TOF samples with the 10-nm CGL using (a) 2-, (b) 1.5-, and (c) 1-µm NPB layer. The intersections of the asymptotes to the plateau (or increasing signal) and the tail section are marked by an arrow together with a text indicating the transit time (*t*_T_). The times required for photovoltage drops to the half of its value at the transit time, denoted as *t*_1/2_ in the main text, are marked with vertically dashed lines. (d) Dispersion parameters of the electron transport at various applied electric fields for the TOF samples with different NPB thicknesses.

**Table S1.** The electron transit times and dispersion parameters at various applied electric fields for the TOF measurements with (w/) the CGL at 532-nm excitation.

| 2-µm NPB w/ CGL @ 532 nm | | | | 1.5-µm NPB w/ CGL @ 532 nm | | | | 1-µm NPB w/ CGL @ 532 nm | | | |
| --- | --- | --- | --- | --- | --- | --- | --- | --- | --- | --- | --- |
| *E* | *t*_T_ (µs) | *t*_1/2_ (µs) | *W* | *E* | *t*_T_ (µs) | *t*_1/2_ (µs) | *W* | *E* | *t*_T_ (µs) | *t*_1/2_ (µs) | *W* |
| 350 kV/cm | 1.16 | 1.19 | 0.03 | 67 kV/cm | 2.85 | - | - | 200 kV/cm | 0.66 | - | - |
| 400 kV/cm | 0.99 | 1.02 | 0.03 | 100 kV/cm | 2.04 | 2.23 | 0.09 | 300 kV/cm | 0.51 | 0.56 | 0.09 |
| 450 kV/cm | 0.86 | 0.89 | 0.03 | 133 kV/cm | 1.56 | 1.70 | 0.08 | 400 kV/cm | 0.47 | 0.50 | 0.06 |
| 500 kV/cm | 0.75 | 0.79 | 0.05 | 167 kV/cm | 1.27 | 1.39 | 0.09 | 500 kV/cm | 0.42 | 0.44 | 0.05 |
|  |  |  |  | 200 kV/cm | 1.06 | 1.15 | 0.08 | 600 kV/cm | 0.37 | 0.38 | 0.03 |

**6. TOF measurement setup for measuring the carrier mobility in NPB/CGL structures**

In the ITO/CGL/NPB/Al structure, the ITO was connected to power supply which provides the electric field for driving the carriers, depending on the polarity (**Figure S6a**). The carriers are only created in the CGL because of the 532-nm excitation source. After the transportation through the NPB, the carriers were collected by the Al electrode and flowed into an oscilloscope. A built-in resistance in the oscilloscope then produces a photovoltage which reflects the transient signal of the carriers. However, in the ITO/NPB/CGL/Al structure, the carriers are generated in the CGL near the Al electrode (**Figure S6b**). The voltage bias must apply to the Al electrode to drive the carrier transporting through the NPB. After the carriers are reaching the ITO electrode, these carriers contribute to the current and flow into the oscilloscope to produce the photovoltage.

**Figure S6.** Measurement setup for TOF prepared by (a) the CGL underneath the NPB and (b) the CGL atop the NPB. The parameters, *V*, *E*, *L*, and *I* denote the voltage, electric field, thickness of NPB, and current, respectively. The solid and open circles denote the electron and hole, respectively. Note that all the structures are not scaled with a real device.

**7. Power-dissipation profiles inside the TOF samples for polymers**

To measure the carrier mobility of polymers, the CGL must be placed onto the polymer to avoid the dissolving problem. In addition, some of the polymers may have an absorption to either 355 or 532 nm, the laser illumination cannot pass through the ITO side in case the polymers absorbs much of the laser power. Alternatively, the top electrode can be replaced by a transparent electrode, such as thin Ag in a thickness of 10-20 nm, thus enabling the laser passing through the transparent electrode (**Figure S7a**). **Figure S7b-d** are the power-dissipation profiles inside the TOF samples for PEOPT, P3HT, and PCBM polymers, respectively..

**Figure S7.** (a) Device configuration for measuring the carrier mobility of polymers. Power-dissipation profiles inside the TOF samples for (b) PEOPT, (c) P3HT, and (d) PCBM polymers.

**8. Thickness dependence of the CGLs in the proposed approach**

To investigate whether the ratios of the NPB to the CGL affect the estimation of carrier mobility, the CGLs with three different thicknesses were used in the TOF samples with a 0.3-µm NPB. **Figures S8a-c** show the transient signals for the TOF samples with a 0.3-µm NPB when the CGL thicknesses of 5, 10, and 15 nm were used, respectively. An excition source of 532 nm was used and the power was appropriately controlled to 30 mW. All the transient signals showed similar profiles independent of the CGL thickness, thus leading to almost indentical hole mobility, as shown in **Figure S8d**. The photovoltage intensity exbhited a decreasing trend with reducing the CGL thickness. This result may indicate that the amount of photo-excited carriers were limited by the CGL thickness. Nevertheless, the estimated hole mobility is almost identical. Therefore, the voltage drop was primarily dominated by the thickness of the NPB instead of the CGL, thus permitting the mesurement of a thinner thickness of the TOF sample with the 5-nm CGL, without concering both quench of photo-generated excitons in the CGL and accuracy in determining the carrier mobility.

**Figure S8.** TOF transient signals of samples for 0.3-µm NPB with (w/) CGL thicknesses of (a) 5, (b) 10, and (c) 15 nm. The insets show the corresponding semi-log plots. The arrows together with texts indicate the transient time. (d) Hole mobility of 0.3-µm NPB with various CGL thicknesses.

**9. Comparison of the transient signals for TOF samples with an unpurified and purified NPB**

**Figure S9** shows the transient signals for TOF samples with an unpurified and purified NPB as the material under test when a CGL with a thickness of 10 nm was used. Both devices showed a perfect plateau, whereas the tail differed considerably, thus indicating the dispersivity of the sample with the unpurified NPB was higher than that of the sample with the purified NPB. The improved dispersivity in the sample with the purified NPB was attributed to the less unexpected doping such as oxygen or defects as compared with unpurified NPB. Therefore, the proposed CGL cannot resolve the issue of unexpected doping effects. A possibility to resolve this problem is to directly remove the unexpected dopants which form defects through the purification of materials under test.

**Figure S9.** Comparison of the transient signals for TOF samples prepared with (a) an unpurified and (b) purified NPB. A CGL with a thickness of 10 nm was used.

**10. Measuring the hole mobility of P3HT by using the proposed CGL under different illumination direction**

To demonstrate the possibility of measuring the carrier mobility of polymers, a TOF sample with a strcuture ITO/P3HT (100 nm)/SubPc:C_70_ (1:5; 5 nm)/Ag (20 nm) was fabricated and tested. **Figures S10a and S10b** show the transient signals for the samples under 355-nm illumination through the ITO and Ag sides, respectively. Although the tail section differed slightly, the transient times can be obtained clearly, as indicated by the arrows. **Figure S10c** shows the hole mobility estimated from the transient signals. The hole mobility was almost identical independent of the illuminating direction, thus indicating that the transparent electrodes are irrelevant in determining the carrier mobility. However, the tail sections of the TOF sample illuminated from the Ag side showed a distinct separation compared with that of the TOF sample illuminated from the ITO side. A possible reason for this observation could have resulted from the unintentional carrier generation at the P3HT/C_70_ interface, which is similar to a bilayer OPV structure. When the 355-nm laser illuminated through the ITO side, the power was firstly absorbed by the P3HT/C_70_ interface and then the SubPc:C_70_ CGL. Holes generated at the P3HT/C_70_ interface transport to the ITO, whereas electrons may recombine with holes originated from the CGL. Because the CGL has a thickness of 5 nm only, holes may be arisen from the P3HT/C_70_ instead of being provided by the CGL. By contrast, when the illumination source passed through the Ag side, the high absorption properties of the CGL enabled absorbing most of the power. Therefore, the hole transport through the P3HT is primarily dominated by holes generated by the CGL, thus leading to the separation of the transient signals, as observed in the NPB samples.

**Figure S10.** TOF transient signals at various electric fields for TOF samples with a 0.1-µm P3HT and 5-nm CGL under 355-nm illumination through the (a) ITO and (b) Ag sides. The intersections of the asymptotes to the plateau and the tail section are marked by an arrow together with a text indicating the transit time (*t*_T_). (c) Hole mobility of the TOF samples. The inset illustrates the device configuration and the direction of illumination.

References

[1] L. A. A. Pettersson, L. S. Roman, O. Inganas, *J. Appl. Phys.* **1999**, *86*, 487-496.

[2] G. F. Burkhard, E. T. Hoke, M. D. McGehee, *Adv. Mater.* **2010**, *22*, 3293-3297.

[3] T.-Y. Chu, O.-K. Song, *Appl. Phys. Lett.* **2007**, *90*, 203512.
